# Transthoracic ultrasound assessment of B-lines for identifying the increment of extravascular lung water in shock patients requiring fluid resuscitation

**DOI:** 10.1186/cc10855

**Published:** 2012-03-20

**Authors:** P Theerawit, N Tomuan, Y Sutherasan, S Kiatboonsri

**Affiliations:** 1 Ramathibodi Hospital, Mahidol University, Bangkok, Thailand

## Introduction

Sonographic B-lines are commonly observed in cases of increasing extravascular lung water (EVLW). These findings became prominent when interstitial and alveolar tissues were filled with fluid [1,2]. Thus, we hypothesized that the increment of sonographic B-lines would be observed when the EVLW increased after fluid resuscitation in shock patients and be associated with the impaired gas exchange.

## Methods

Transthoracic portable ultrasound before and after fluid resuscitation was performed. Patients with pleural disease were excluded. The B-lines were measured in 23 lung zones. The total numbers of B-lines seen in each patient were counted as the total B-line score (TBS). The primary outcome was to demonstrate the increase of TBS after fluid resuscitation. The secondary outcome was to examine the magnitude of the incremental number of TBS.

## Results

Twenty patients were enrolled in this study. All patients had septic shock. Six of all had lung involvement. Twelve patients received mechanical ventilation. The mean of net fluid balance was +2,228 ± 1,982 ml and the mean of duration between two ultrasound measurements was 31 ± 13 hours. The means of TBS at pre and post fluid therapy were 37 ± 26 and 64 ± 29 respectively (*P *< 0.0001, 95% CI 13.47 to 33.67). This increase was found in all areas of measurement. In particular, the number of B-lines measured at the anterior axillary line area very well correlated to the TBS (*r *= 0.90, *P *< 0.01) and its increment had reverse correlation to the PaO_2_/FiO_2 _ratio (*r *= 0.704, *P *< 0.05). The volume of fluid per one B-line increasing was 119 ± 134 ml. The interobserver reliability between two ultrasound readers was very high (*r *= 0.92, *P *< 0.01). The changing of TBS did not correlated to that of the chest radiologic score for EVLW assessment (*r *= 0.002, *P *> 0.05). There was no linear correlation observed between net fluid balance and total number of increasing B-lines.

## Conclusion

The number of B-lines definitely increased after fluid resuscitation in shock and correlated to the deterioration of pulmonary gas exchange. These data support the benefit of transthoracic portable ultrasound for assessment of the increment of EVLW in shock patients receiving fluid resuscitation.

## References

[B1] Bouhemad B Zhang M Lu Q Clinical review: Bedside lung ultrasound in critical care practice Crit Care 2007 11 205 10.1186/cc5668 17316468 PMC2151891

[B2] Soldati G Copetti R Sher S Sonographic interstitial syndrome: the sound of lung water J Ultrasound Med 2009 28 163 174 19168766 10.7863/jum.2009.28.2.163

